# A Point Cloud Graph Neural Network for Protein–Ligand Binding Site Prediction

**DOI:** 10.3390/ijms25179280

**Published:** 2024-08-27

**Authors:** Yanpeng Zhao, Song He, Yuting Xing, Mengfan Li, Yang Cao, Xuanze Wang, Dongsheng Zhao, Xiaochen Bo

**Affiliations:** 1 Academy of Military Medical Sciences, Beijing 100850, China; zyp182531903@163.com (Y.Z.); hes1224@163.com (S.H.); li.mengfan@outlook.com (M.L.); cho.yang@foxmail.com (Y.C.); xuanze.wang@foxmail.com (X.W.); 2 Defense Innovation Institute, Beijing 100071, China; sukier1@126.com

**Keywords:** drug discovery, deep learning, protein–ligand binding site, point cloud, structure representation, graph neural network

## Abstract

Predicting protein–ligand binding sites is an integral part of structural biology and drug design. A comprehensive understanding of these binding sites is essential for advancing drug innovation, elucidating mechanisms of biological function, and exploring the nature of disease. However, accurately identifying protein–ligand binding sites remains a challenging task. To address this, we propose PGpocket, a geometric deep learning-based framework to improve protein–ligand binding site prediction. Initially, the protein surface is converted into a point cloud, and then the geometric and chemical properties of each point are calculated. Subsequently, the point cloud graph is constructed based on the inter-point distances, and the point cloud graph neural network (GNN) is applied to extract and analyze the protein surface information to predict potential binding sites. PGpocket is trained on the scPDB dataset, and its performance is verified on two independent test sets, Coach420 and HOLO4K. The results show that PGpocket achieves a 58% success rate on the Coach420 dataset and a 56% success rate on the HOLO4K dataset. These results surpass competing algorithms, demonstrating PGpocket’s advancement and practicality for protein–ligand binding site prediction.

## 1. Introduction

Binding sites on the surface of three-dimensional (3D) proteins often appear as deep grooves or tunnels that accommodate small-molecule drugs or ligands. These regions, known as binding pockets or cavities, are crucial for protein–molecule interactions. Accurately detecting these binding sites is paramount in drug discovery and design [1,2,3]. By thoroughly understanding the binding sites, molecules can be precisely designed to bind with proteins, thereby increasing the efficacy of the drug, minimizing side effects, and making the treatment safer and more effective [4,5].

Over the last few decades, many methods have been proposed to identify protein binding sites, which can be classified into three categories: geometry-based, template-based, and energy-based methods [6]. Geometry-based methods predict binding sites based on the geometrical features of the protein surface and rank candidate sites by their draggability [7,8,9]. Template-based methods search for the most similar proteins in a database and assign the binding sites of the hit proteins to the query protein, requiring a large number of protein structures with known binding sites [10]. Energy-based methods identify ligands with the minimum interaction energy needed to bind to a protein, typically requiring multiple matches and numerous ligand templates [11,12,13].

Various computational tools have been developed to predict potential ligand-binding sites. Fpocket version 2.0 [7] is an open-source software package based on Voronoi partitioning and alpha-sphere concepts, suitable for a wide range of protein structures. It offers functionalities including cavity identification, benchmarking, and calculation of cavity descriptors. MetaPocket 2.0 (MPK2) [14] combines eight different pocket detection tools to predict drug-binding sites on protein surfaces. MPK2 enhances prediction robustness by integrating the top three predictions from each method, clustering them into “meta-pockets”, and reordering the results. The bSiteFinder server, developed by Gao et al. [15], uses homology indexing, chain length indexing, complex stability assessment, and optimized multi-template clustering to significantly improve prediction performance. In 2022, Wang et al. updated the CavityPlus 2022 platform [16,17,18], a comprehensive web server for detecting and characterizing protein cavities. Each of these tools is unique and suitable for different applications. However, these methods have limitations, such as difficulty in identifying dynamic pockets or sensitivity issues with irregularly shaped cavities.

With the continuous accumulation of protein sequences and 3D structures, the continuous updating and optimization of artificial intelligence (AI) algorithms, and the increasing computational power of computer hardware, machine learning, particularly deep learning models, has been developed to predict the location of binding sites. Machine learning-based methods are mainly classified into sequence-based and structure-based methods. Sequence-based methods rely on the amino acid sequence of a protein as the primary input, analyzing sequence information to identify binding sites [19,20,21]. Commonly used protein sequence features in protein–ligand binding site prediction include amino acid composition, physicochemical properties of amino acids (e.g., hydrophobicity, polarity, charge, and solvent accessibility), and evolutionary conservation profiles. The latter includes the frequency of amino acids observed in homologous sequences, with commonly used features such as position-specific scoring matrix (PSSM) [22] and hidden Markov models matrix (HMM) [23].

Structure-based methods rely on the three-dimensional structural information of proteins to identify protein–ligand binding sites. Machine learning methods typically use manually extracted one-dimensional (1D) representations of protein structures, incorporating a range of spatial and geometric features [24]. For example, the curvature distribution of the protein surface is a commonly used feature to help understand the shape and accessibility of potential binding sites. Additionally, structure features such as the state of secondary structure elements, residue solvent accessibility, torsion angles, and bond angles have been widely used to capture the conformational properties necessary for ligand binding. However, these manually extracted features have the disadvantage of relying too much on expert knowledge. With the development of deep learning techniques, automatic feature learning methods are becoming more mainstream due to their higher efficiency, generalizability, and performance. In recent years, there has been a rapid rise in geometric deep learning (GDL), an umbrella term encompassing emerging techniques for generalizing neural networks to Euclidean and non-Euclidean domains (e.g., graphs, point clouds, meshes, or string representations) [25]. GDL has been shown to greatly outperform manually designed features in feature extraction [26]. For example, in the DELIA framework [27], proteins are represented as two-dimensional amino acid distance matrices, encoding pairwise distances between amino acid residues to capture their spatial relationships in the 3D structure. A convolutional neural network (CNN) processes these matrices. DeepSite and DeepSurf represent protein surfaces as 3D grids, and then the 3D grids are divided into multiple subgrids [28], characterized by their coordinates and potentially additional features such as amino acid type or physicochemical properties, which 3DCNN is often used to process these features [29,30]. In addition, Kalasanty and PUResNet [31,32] represent the protein structure as a 3D image for binding site prediction using U-Net-based networks [33]. GLPocket [34] uses the Lmser architecture [35,36] to capture a multi-scale representation of the protein to predict the binding site.

Unlike traditional grid or image representations, point cloud representations intuitively capture the geometric features of protein surfaces, such as surface concavity and curvature [37]. In addition, point clouds can flexibly adapt to the various complex shapes of protein surfaces without being limited to a fixed resolution. This flexibility has made them highly successful in tasks such as protein–ligand affinity and protein–protein binding site prediction [38,39,40]. Graph neural networks (GNNs) [41] are a crucial component of GDL. GNNs capture local structural information between points by constructing neighborhood graphs, which helps to identify specific structural features near binding sites. More importantly, a GNN can better handle the rotational and translational invariance of proteins, which is essential for accurate binding site prediction [42,43,44].

In this study, we propose a protein–ligand binding site prediction framework based on a point cloud graph neural network, PGpocket. Initially, the protein surface is encoded as a point cloud to obtain a fine-grained representation of the protein features, allowing the computation of geometric and chemical characteristics for each point. We then construct a point cloud graph based on the distances between points on the protein surface and use a point cloud graph neural network to learn the protein surface information and predict binding sites. PGpocket was trained on nearly 5000 protein datasets from the scPDB database [45] and evaluated on two independent test sets. The results show that the model accurately captures binding site features and outperforms two other competing algorithms. The source code is available at https://github.com/zhaoyanpeng208/PGpocket, accessed on 3 August 2024.

## 2. Results and Discussion

### 2.1. Overview

**Figure 1:**
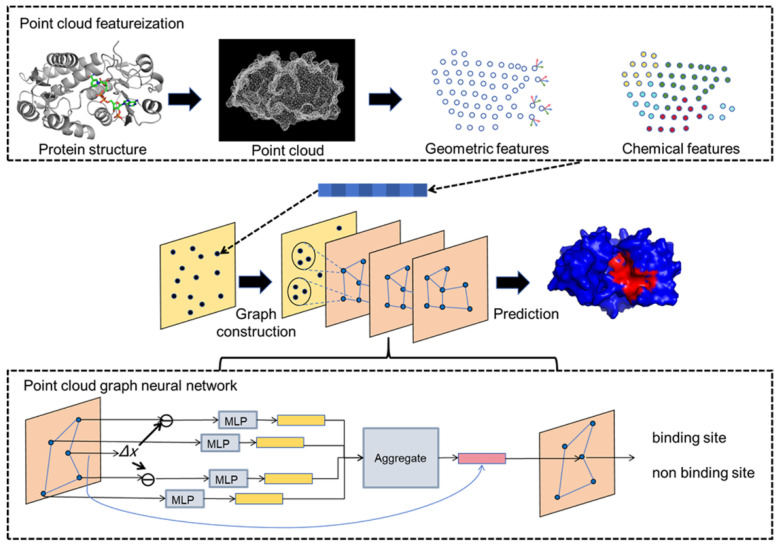
The framework of PGpocket. The framework consists of two core components: point cloud featurization and point cloud graph neural networks. For any input protein structure, the protein surface is first abstracted into a point cloud representation, and geometric and chemical features are extracted for each point. Subsequently, the points of the point cloud are downsampled, and connecting edges are constructed based on the distances between the points to form a point cloud graph. This point cloud graph serves as the input to the point cloud GNN. The point cloud GNN updates the node features by aggregating features from neighboring nodes. Ultimately, the network outputs the probability of each node being a binding site.

The PGpocket framework is designed for efficient and accurate prediction of protein–ligand binding sites. As shown in Figure 1, PGpocket is divided into two modules: point cloud featurization and a point cloud GNN. In the point cloud featurization module, the protein surface is transformed into a point cloud, a series of discrete spatial coordinate points, using a continuous geometric representation. For each point in the point cloud, chemical and geometric features are calculated to form its initial representation. Geometric features include mean curvature and Gaussian curvature, reflecting local shape, and chemical features are based on the statistical aggregation of surrounding atom types, reflecting the chemical microenvironment. In the point cloud GNN module, we benefit from the PointGNN algorithm [46], a graph neural network designed for object detection, which is capable of efficiently processing point cloud data. First, the edges of the graph are constructed based on Euclidean distances between points in the point cloud to form the point cloud graph. To manage computational complexity due to the potentially large number of points, a downsampling strategy is used to reduce the number of points while retaining key information to avoid excessive loss. The downsampled point cloud graph is then input into the GNN, which updates each node’s representation by aggregating feature vectors of neighboring nodes to capture higher-level spatial chemical associations. Auto-registration is also utilized to overcome translational variance, ensuring robustness to positional variations of proteins and improving prediction accuracy. Finally, the likelihood score for each point being a binding site is output, representing the probability that the point belongs to a binding site. Points predicted as binding sites are clustered using the clustering algorithm to identify continuous or aggregated sets of points that constitute the protein’s binding pocket.

### 2.2. Evaluation Metrics

From a three-dimensional structural perspective, binding sites are grooves or pockets in the spatial conformation of a protein that can accommodate specific ligands. There are two primary ways to define these binding sites accurately: (1) Residue-level approach: This method considers binding sites as those amino acid residues with at least one non-hydrogen atom within the van der Waals radius of the ligand’s non-hydrogen atoms plus 0.5 Å. This approach is widely used in official CASP/CAMEO assessments [10,27]. (2) Protein surface-level approach: This method defines binding sites as points or grids on the protein surface. Points or grids are considered part of the binding site if they are within 4 Å of the geometrical center of the binding site. This threshold is based on common practice in binding site literature, and predictions are deemed successful if the predicted points are closer to the actual binding site than this threshold [24,30].

For PGpocket, we chose the second definition method (protein surface-based method) to define the binding site. This choice aligns with the characteristics of the PGpocket method, where each sample represents a point on the protein surface. This approach simplifies the prediction scale and facilitates model training and validation.

When evaluating the performance of a binding site prediction model, the use of two metrics, distance center center (DCC) and discretized volume overlap (DVO), provides a comprehensive view of the model’s accuracy and prediction quality. Below is an explanation of these two metrics and how to use DCC to calculate F1-scores [31,32]. It is important to note that the definitions of TP, FP, FN, and F1-score used in this work follow the definitions set by PUResNet [32]. Unlike the definitions used in COACH [10], where the F1-score is defined at the amino acid level, the F1-score in this work is defined at the protein pocket level. Therefore, direct comparisons based on these metrics between PGpocket and COACH cannot be made.

#### 2.2.1. Distance Center Center (DCC)

DCC measures the distance between the center of the predicted binding site and the center of the actual binding site or ligand. A prediction is considered correct if this distance is less than or equal to 4 Å. The success rate, which indicates the proportion of correct predictions, is defined as follows:

(1) $$Success\;Rate=\frac{Number\;of\;sites\;having\;DCC\leq 4\;Å}{Total\;number\;of\;sites}$$

#### 2.2.2. Discretized Volume Overlap (DVO)

DCC considers only the position of the center and ignores the volume and shape of the binding site. DVO compensates for this by calculating the ratio of the intersection to the union of the volumes of the predicted binding site ($V_{pbs}$) and the actual binding site ($V_{abs}$). The DVO formula is the following:

(2) $$DVO=\frac{V_{pbs}\cap V_{abs}}{V_{pbs}\cup V_{abs}}$$

#### 2.2.3. F1-Score

The F1-score is a reconciled average of precision and recall for unbalanced datasets. In this scenario, we define the following: 

True Positive (TP): The predicted binding site DCC is less than or equal to 4 Å.

False Positive (FP): The predicted binding site DCC is greater than 4 Å.

False Negative (FN): There is no predicted binding site, but one is present.

Since all protein structures in the dataset have at least one binding site, there are no true negative cases. The F1-score is calculated using the formula:

(3) $$F1\;score=2\times \frac{Precision\times Recall}{Precision+Recall}$$

where

(4) $$Precision=\frac{TP}{TP+FP}$$

(5) $$Recall=\frac{TP}{TP+FN}$$

### 2.3. Performance on the scPDB Dataset

Protein structures with protein–ligand binding sites were collected from the scPDB database [45] (see Method for details). The dataset was strictly divided into training, validation, and test sets in the ratio of 8:1:1. The models were subjected to training and performance validation on the training and validation sets to ensure their generalization ability and prediction accuracy. Finally, their performance was tested on an independent test set containing 482 proteins with a total of 529 binding sites (some proteins have multiple binding sites). 

To objectively assess model performance, we systematically compared PGpocket with existing frameworks COACH [10], Kalasanty [31], PUResNet [32], and P2Rank [24], and the results are shown in Table 1 and Supplementary Table S1. It is important to note that COACH uses a residue-level definition for binding site prediction. To enable a comparison, we first used COACH to determine the binding residues. Subsequently, we calculated the DCC using the C_α_ atom coordinates of the binding residues. Additionally, we computed the DVO based on the volume of atoms contained within the binding residues. These calculations may potentially lead to a decrease in COACH’s performance due to the conversion from residue-level predictions to protein surface-level evaluations. 

As shown in Table 1 and Supplementary Table S1, among the protein samples in the test set, COACH identified 298 binding sites but missed binding sites in 34 protein samples (approximately 7% of the total) with an F1-score of 0.721. Kalasanty identified 270 binding sites, failing to find binding sites in 28 protein samples (approximately 6% of the total), resulting in an F1-score of 0.676. PUResNet successfully identified 322 binding sites, yet it did not identify binding sites in 48 samples (10% of the total), yielding an F1-score of 0.757. P2Rank accurately located 340 binding sites and missed binding sites in 25 samples (5% of the total) with an F1-score of 0.783. In contrast, PGpocket successfully identified 350 binding sites and missed binding sites in only 13 samples, representing 2.6% of the total, achieving an F1-score of 0.796. This underscores the significant advantage of the PGpocket method in binding site prediction.

**Figure 2:**
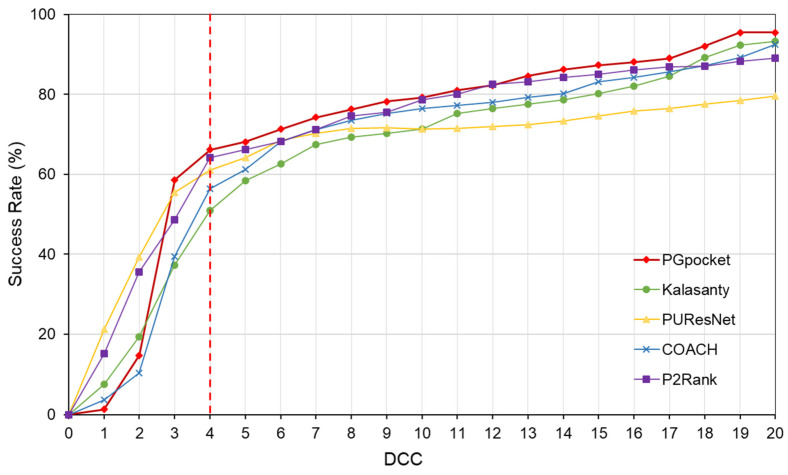
Success rate plot of different DCC values for PGpocket and the baselines on scPDB dataset. The red dotted line represents the performance of the 5 frameworks when DCC is less than or equal to 4 Å.

**Figure 3:**
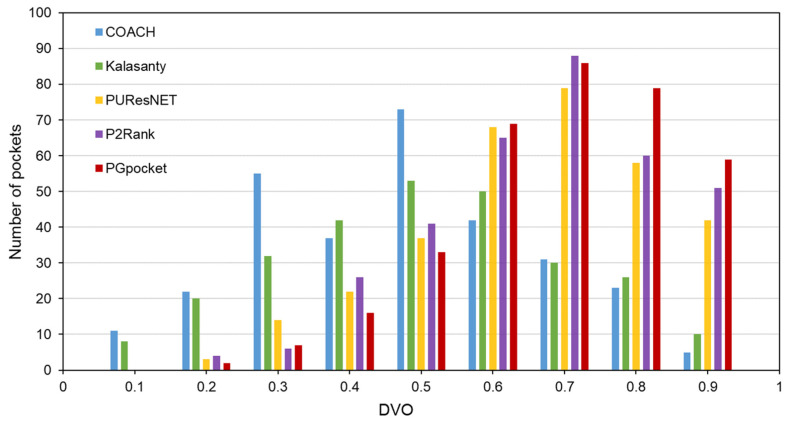
Histogram of DVO values for PGpocket and the baselines on scPDB dataset.

In terms of success rate, PGpocket performs exceptionally well on the test set with a rate of 66.2%, compared to 56.4% for COACH, 51% for Kalasanty, 61% for PUResNet, and 64.2% for P2Rank (Figure 2, Supplementary Table S2). Additionally, PGpocket excelled in the DVO metric, achieving a mean value of 0.65 (Figure 3, Supplementary Table S3). This value is significantly higher than those of the four baseline methods, confirming PGpocket’s superior accuracy in predicting the volume overlap of binding sites.

In summary, compared to COACH, Kalasanty, PUResNet, and P2Rank, PGpocket not only excels in binding site identification accuracy but also demonstrates higher accuracy in predicting the volume and shape of binding sites.

### 2.4. Performance on Two Independent Test Sets

On independent test sets Coach420 and HOLO4K [24], we thoroughly evaluate the prediction performance of PGpocket and analyze the results in comparison with four existing frameworks, COACH, Kalasanty, PUResNet, and P2Rank. Similarly, to evaluate COACH, we continued the processing from the previous section. We re-emphasize that this approach may lead to some decreases in the performance of COACH, as errors may be introduced during the conversion from residue-level prediction to protein surface-level evaluation. 

**Figure 4:**
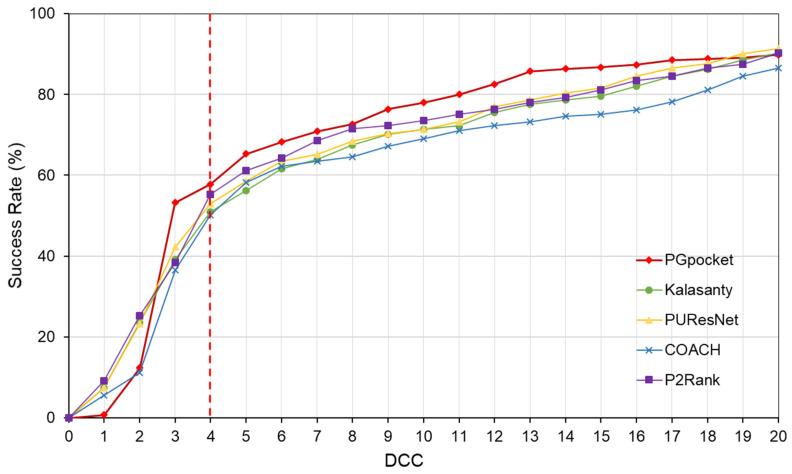
Success rate plot of different DCC values for PGpocket and the baselines on Coach420 dataset. The red dotted line represents the performance of the 5 frameworks when DCC is less than or equal to 4 Å.

**Figure 5:**
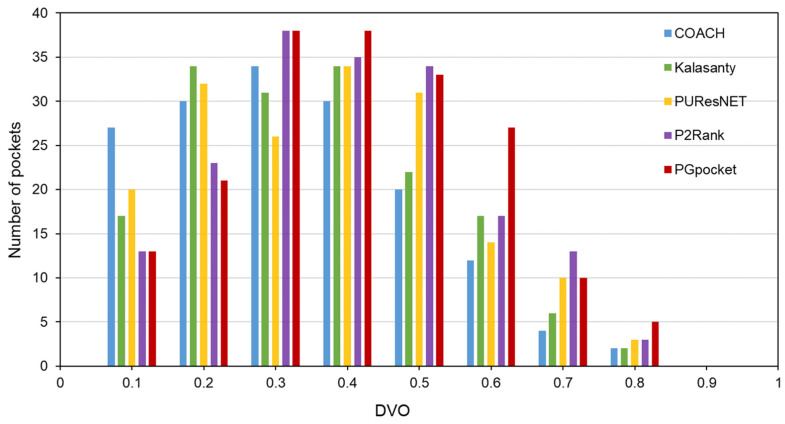
Histogram of DVO values for PGpocket and the baselines on Coach420 dataset.

The Coach420 dataset consists of 298 protein structures with a total of 318 binding sites. As shown in Table 2 and Supplementary Table S4, PGpocket missed the fewest binding sites, only 13, and achieved an F1-score of 0.733. In comparison, COACH missed 28 binding sites and achieved an F1-score of 0.667; Kalasanty missed 26 and achieved an F1-score of 0.675; PUResNet missed 19 and achieved an F1-score of 0.694; and P2Rank missed 20 binding sites and achieved an F1-score of 0.710. Figure 4 and Supplementary Table S5 illustrate the success rates of the frameworks under different DCCs. COACH has the lowest success rate at 50.1%, followed by Kalasanty at 51.0%, PUResNet at 52.8%, and P2Rank at 55.2%. In contrast, PGpocket exhibits the highest success rate at 57.8%, indicating superior accuracy in predicting binding sites. Figure 5 and Supplementary Table S6 report the DVO values of the frameworks, with PGpocket leading with an average DVO of 0.35. The DVO averages for the other models are 0.28 for COACH, 0.30 for Kalasanty, 0.32 for PUResNet, and 0.34 for P2Rank. This suggests that PGpocket is more effective at predicting binding site volume overlap than the other models, demonstrating the highest accuracy in this metric.

**Figure 6:**
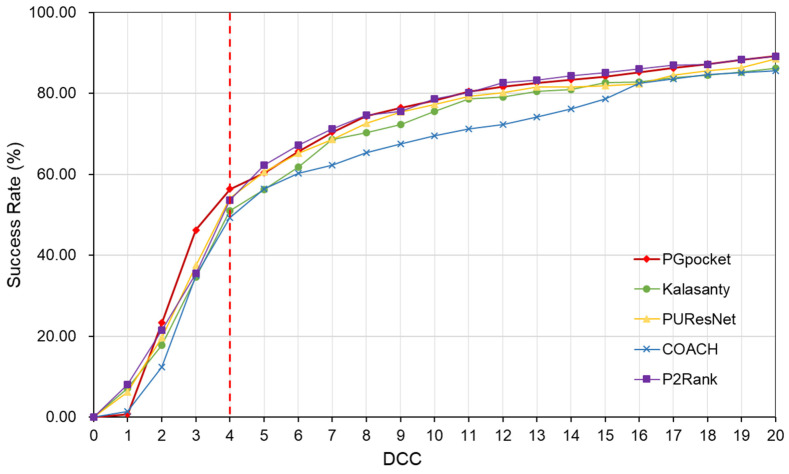
Success rate plot of different DCC values for PGpocket and the baselines on HOLO4K dataset. The red dotted line represents the performance of the 5 frameworks when DCC is less than or equal to 4 Å.

**Figure 7:**
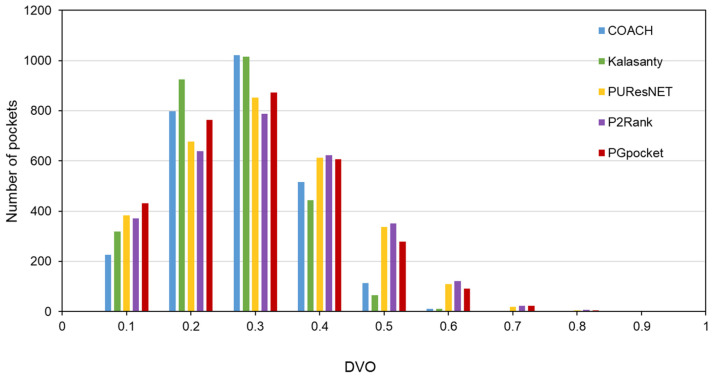
Histogram of DVO values for PGpocket and the baselines on HOLO4K dataset.

The HOLO4K dataset is considerably larger, consisting of 4055 protein structures with a total of 5444 binding sites. As shown in Table 2 and Supplementary Table S4, PGpocket fails to identify the fewest number of binding sites, missing 248 and achieving an F1-score of 0.721. PUResNet, on the other hand, fails to identify the most, with 408 missed sites and achieving an F1-score of 0.710. Kalasanty, COACH, and P2Rank miss 325, 364, and 298 binding sites, with F1-scores of 0.675, 0.660, and 0.698, respectively. In terms of success rate, as depicted in Figure 6 and Supplementary Table S7, PGpocket exhibits the highest success rate at 56.3%, indicating its superior accuracy in predicting binding sites. COACH has the lowest success rate at 49.3%, while Kalasanty, PUResNet, and P2Rank have success rates of 51.0%, 54.0%, and 53.6%, respectively. Regarding DVO (Figure 7, Supplementary Table S8), P2Rank performs the best with an average DVO of 0.26, followed by PUResNet at 0.25. PGpocket’s average DVO is 0.24, slightly lower than P2Rank but still competitive. COACH and Kalasanty have DVO averages of 0.23 and 0.21, respectively.

Overall, for both the Coach420 and HOLO4K datasets, PGpocket demonstrates advantages over COACH, Kalasanty, PUResNet, and P2Rank, confirming its excellent performance in the task of protein–ligand binding site prediction.

### 2.5. Ablation Experiments

To test the effectiveness of PGpocket’s downsampling in reducing computation time while maintaining model performance, we compared the performance of three different model configurations on the scPDB dataset. These configurations included: (1) a model trained using all points in the point cloud, (2) a model using downsampling but no feature aggregation, and (3) a model using downsampling with feature aggregation. We compared their performance and the time required for prediction.

As shown in Table 3 and Supplementary Table S9, the model using all points achieved an F1-score of 0.805 with a success rate of 0.674, but the average time to make predictions on individual proteins was 25.36 s. The model with downsampling but no feature aggregation achieved an F1-score of 0.760 with a success rate of 0.612 and an average time of 12.56 s. The model with downsampling and feature aggregation achieved an F1-score of 0.796 with a success rate of 0.662 and an average time of 14.65s.

Overall, the model using all points provided the best prediction quality at the expense of computational efficiency. The model with downsampling but no feature aggregation performed the best in terms of computational efficiency but declined in terms of prediction quality. The model with downsampling and feature aggregation provides a compromise that improves computational efficiency while ensuring relatively high prediction quality.

### 2.6. Case Study at ATP Binding Site Prediction

ATP (adenosine triphosphate), a key biomolecule, plays a fundamental role in cellular energy metabolism and signal transduction. When ATP binds to proteins, this interaction often triggers changes in protein conformation or regulates its activity, affecting a wide range of biological processes, such as signaling cascade reactions, cellular motility, protein synthesis, and catabolism. Therefore, an in-depth understanding of ATP–protein binding mechanisms, especially the identification of ATP binding sites, is important for resolving complex regulatory networks and metabolic pathways in the cell.

**Figure 8:**
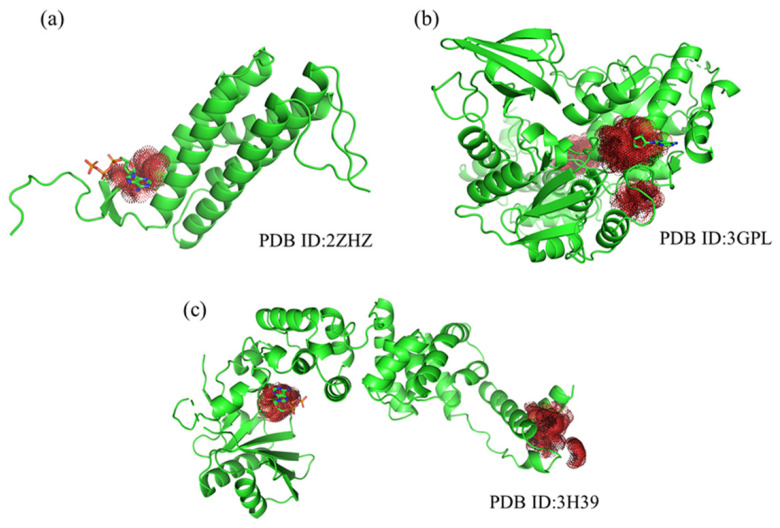
Protein structures (2ZHZ, 3GPL, 3H39) from Coach420, showing predicted binding sites by PGpocket (Red Region). (**a**), PGpocket prediction results on structure 2ZHZ. (**b**), PGpocket prediction results on structure 3GPL. (**c**), PGpocket prediction results on structure 3H39.

To evaluate the efficacy of PGpocket in predicting ATP binding sites, we specifically selected three proteins (PDB ID: 2ZHZ, 3H39, 3GPL) known to bind ATP from the Coach420 dataset and visualized their prediction results (Figure 8). For all three protein structures, PGpocket accurately identified the ATP binding sites, demonstrating its reliability and accuracy in predicting ATP binding sites. On protein structure 2ZHZ, PGpocket identified only one binding pocket, which coincided with a known ATP binding site. For the structures 3GPL and 3H39, PGpocket predicted two and three possible binding pockets, respectively, with the top-ranked predicted binding sites aligning with the actual ATP binding sites. This further verified PGpocket’s efficiency and accuracy in identifying ATP binding sites. In contrast, Kalasanty failed to predict any ATP binding site when facing these three structures, revealing its limitation in this kind of prediction task. While PUResNet, PRANK, and COACH successfully predicted ATP binding sites on 3H39 and 3GPL, it made a prediction error on the 2ZHZ structure, suggesting that the prediction accuracy of these methods could be improved.

In summary, PGpocket demonstrated excellent ability in predicting ATP binding sites, not only achieving high accuracy on three representative samples but also highlighting its significant advantages in the field of ATP binding site prediction in comparison with Kalasanty and PUResNet. This result highlights the potential of PGpocket as a tool for protein–ligand binding site prediction, which is expected to be a powerful aid, especially in studies involving ATP-related biological processes.

## 3. Materials and Methods

### 3.1. Datasets Preparation

In this study, the dataset used in PUResNet [32] was used to train and evaluate our model, which is a subset of scPDB (http://bioinfo-pharma.u-strasbg.fr/scPDB/, accessed on 2 August 2024) and includes 5020 protein–ligand complex structures obtained from 1243 protein families. Removing complex structures that could not be point-clouded, we finally collected a total of 4895 protein–ligand complex structures. To avoid data leakage during model validation, we made all the structures from the same family appear in the same dataset in the process of dividing them into training set, validation set, and test set in the ratio of 8:1:1.

In addition, we selected the COACH420 and HOLO4K datasets [24] as independent test sets. COACH420 consists of 420 protein–ligand complex structures. We performed a de-redundancy process on the dataset by removing the structures that appeared in the training set, in which 122 protein–ligand complex structures were removed, and finally 298 complex structures were retained. For HOLO4K, we performed the same operation and retained 4055 complex structures.

### 3.2. Data Representation

To generate a continuous geometric representation of the protein, MaSIF [40] was used to generate a point cloud representation of the protein surface and to characterize the points in the point cloud. Specifically, given a protein structure, calculate its discretized molecular surface (excluding surface solvents) and assign geometric and chemical features to each point (vertex) in the mesh. For chemical features, firstly each point was encoded as a one-hot vector according to the chemical type in the list [C, H, O, N, S, Se]. Secondly, the atom types and inverse distances to the surface points were nonlinearly and batch normalized by a small MLP with 6 hidden units, ReLU. Finally, the contributions of the 16 atoms closest to the surface point were summed and linearly transformed to generate a vector of 6 scalar features. For the geometric features, we represent the surface of the protein as a directed point cloud $P=\left\{p_{1},\cdots ,p_{N}\right\}\;$with a unit normal vector of $\left[n_{1},\cdots ,n_{N}\right]$. The normals ($n_{i}$) are used to compute the mean curvature and Gaussian curvature at five scales σ from 1 Å to 10 Å. The mean curvature and Gaussian curvature at the five scales are concatenated to form a 10-dimensional vector as the geometrical feature of the point. Finally, the chemical features were concatenated with the geometric features to create a 16-dimensional full feature vector.

### 3.3. Model Construction

To construct this model, we refer to PointGNN [46], a graph neural network algorithm designed for object detection. PointGNN has demonstrated a strong performance in processing point cloud and can identify and localize objects from point clouds efficiently. Given the success of PointGNN, we have borrowed its core ideas and techniques and applied them to the task of protein–ligand binding site prediction.

Based on the constructed dataset and data representation method above, we constructed the GNN model. The model takes the point cloud graph as input and outputs whether each vertex is a binding site or not. The overall architecture of the method consists of two parts: (a) graph construction; (b) GNN for point cloud learning.

### 3.4. Graph Construction

Formally, we define a point cloud of N points as a set $P=\{p_{1},\cdots ,p_{N}\}$, where $p_{i}=(x_{i},h_{i})$ represents one of the points with three-dimensional coordinates $x_{i}\in R^{3}$, and $h_{i}\in R_{m}$ is a vector of length m representing the point attributes. In this study, $h_{i}\;$refers to the combination of chemical and geometric features in 16 dimensions. Given a point cloud P, we construct a graph G=(P,E) by connecting a point to its neighbors within a fixed radius r with P as the vertex.

(6) $$E=\{(p_{i},p_{j})|{\left\|x_{i}-x_{j}\right\|}_{2}<r\}$$

In practice, a point cloud typically consists of tens of thousands of points. Constructing a graph with all points as vertices imposes a large computational burden. Therefore, we use voxel-downsampling point cloud (P) for graph construction. To be noted that voxels here are only used to downsample the density of the point cloud and are not used as a representation of the point cloud. We still use the graph to represent the downsampled point cloud. To preserve the information inside the original point cloud, we encode the dense point cloud in the initial state values${\;h}_{i}$ of the vertices. Specifically, we search for the original points within the $r_{0}$ radius of each vertex and use a neural network on the set to extract their features. We embed the feature values and relative coordinates of the points with an MLP, and then aggregate them via the Max function. We use the resulting features as initial state values for the vertices. After completing the construction of the graph, we process the graph using a GNN.

### 3.5. Graph Neural Network

We design a GNN to refine the representation of vertices to better aggregate information about the vertex’s neighbors. Specifically, we use the states of the neighboring nodes to refine the states of the nodes:

(7) $$h_{i}^{k+1}=g^{k}(\varphi \left(\left\{f^{k}\left(x_{j}-x_{i},h_{j}^{k}\right)|\left(i,j\right)\in E\right\}\right),h_{i}^{k})$$

where *h* represents the node features, and *k* represents the number of iterations, i.e., the number of neural network layers. x is the Cartesian coordinates of the node. $f^{k}(.)$ calculates edge features by two node features between edges. φ(.) is an aggregation function that aggregates the edge features for each vertex. g(.) updates the node features using the aggregated edge features. Here, we use the relative coordinates of the neighbors as an input of $f^{t}(.)$ for edge feature extraction. The relative coordinate is translation invariant to the global displacement of the point cloud. While it remains sensitive to changes in neighboring nodes. Specifically, when a vertex adds a small translation, the local structure of its neighbors remains similar. However, the relative coordinates of the neighbors all changed, which will cause the input variance of $f^{t}\left(.\right)$ to increase. To reduce the translation variance, we align the coordinates of the neighbors based on their structural features rather than the centroid coordinates. Since the centroid already contains some structural features from the previous iteration, we can use it to predict an alignment offset and propose an automatic alignment mechanism:

(8) $$∆x_{i}^{k}=\omega^{k}(h_{i}^{k})$$

(9) $$h_{i}^{k+1}=g^{k}(\varphi (\left\{f\left(x_{j}-x_{i}+∆x_{i}^{k},h_{i}^{k}\right)\right\}),h_{i}^{k})$$

where $∆x_{i}^{k}$ is the coordinate offset from the vertex coordinates, $\omega^{k}\left(.\right)\;$calculating the offset using the centroid of the previous iteration.

$f^{t}(.)$, $g^{t}\left(.\right),\;$and $h^{t}(.)$ are modeled using an *MLP*, and residual connections are added to $g^{t}(.)$. We choose the Max function as φ(.) because of its robustness. In each iteration, the graph neural network is iterated by the following equation:

(10) $$∆x_{i}^{t}={MLP}_{\omega }^{t}(h_{i}^{t})$$

(11) $$e_{ij}^{t}={MLP}_{f}^{t}([x_{j}-x_{i}+∆X_{i}^{t}{,h}_{j}^{t}])$$

(12) $$h_{i}^{t+1}={MLP}_{g}^{t}(Max(\{e_{ij}|(i,j)\in E\}))+h_{i}^{t}$$

A different *MLP* is used for each iteration k, and parameters are not shared between iterations. After T iterations of the graph neural network, we use the vertex representation to predict whether a vertex is a binding site. A classification *MLP* is used to compute the classification probability.

### 3.6. Implementation Details

PGpocket has been implemented in Python 3.8 and PyTorch 2.1.0 as well as functions in PYG 2.4.0, Scikit-learn 1.3.2, Numpy 1.24.1, Pandas 2.1.3, and biopython 1.81. In the point cloud downsampling operation, we set the voxel size to be used to reduce the point cloud density and aggregate the features with radius r0=2 Å. For the initial features, we use an MLP of size (16,300) to obtain the embedding of each point in the point cloud. Subsequently, the graph is constructed with radius r=4 Å, and the maximum number of initial graph edges is set to 32. We set the number of GNN layers to three, where the size of ${MLP}_{f}\;$and ${MLP}_{g}\;$is (300,300), and the size of ${MLP}_{w}$ is (64,3). Finally, a 4-layer MLP is used to classify each point.

The batch size was set to 4, the Adam optimizer was used with a learning rate of 5 × 10^−4^, and a weight decay of 1 × 10^−5^. We allowed the model to run for most 50 epochs.

### 3.7. Clustering Method

In the prediction process, for a point in the point cloud, if the output probability is greater than 0.5, it is considered to be a predicted positive sample. However, the number of samples predicted to be binding sites is often in the hundreds, and some of the predicted sites are close enough to form binding regions in the protein structure, which implies that such predicted sites should be aggregated into a binding pocket. Therefore, we use the OPTICS clustering algorithm [47] to solve them. In this way, hundreds of binding sites in a structure can be clustered into several binding pockets. We use the clustered binding pockets as the final predictions for the calculation of DCC and DVO values.

Following the clustering of predicted binding pockets, we rank them according to the sum of the probabilities of the points within each cluster. Clusters with a higher sum of point probabilities are ranked higher, indicating a stronger likelihood of being a true binding site. For the rank cut-off, we used the number of actual binding sites as the cut-off for prediction. Specifically, if the protein has n known binding sites, we selected the top-n ranked predicted binding sites to calculate DCC and DVO.

### 3.8. Baselines

#### 3.8.1. PUResNet

PUResNet [32] applies the ResNet architecture to extract embeddings of proteins. Specifically, the protein structure is input into PUResNet as a 3D image of shape (36 × 36 × 36 × 36 × 18), and the output is a single channel of the same shape as the input (i.e., 36 × 36 × 36 × 1), where each voxel (point in 3D space) in the output has a probability of determining whether or not the voxel is a cavity.

#### 3.8.2. Kalasanty

Kalasanty [31] is based on U-Net18 and uses the model for the binding pocket detection problem. The model takes a protein structure as input, automatically converts it to a 3D mesh with features, and outputs a probability density; i.e., each point in 3D space is assigned a probability of being part of a pocket.

#### 3.8.3. COACH

COACH [10] combines two complementary techniques: the TM-SITE method, which is based on binding-specific substructure comparisons, and the S-SITE method, which uses sequence profile comparison. Both methods aim to improve the accuracy and coverage of predictions. TM-SITE identifies potential binding sites by comparing low-resolution structural models of target proteins with structural templates of known binding sites. S-SITE uses sequence profile comparison to identify evolutionarily conserved binding sites.

It is important to note that the output of these methods labels which amino acids in the protein are binding sites, rather than binding pockets. Therefore, in the comparisons presented here, the binding amino acids are first identified using this algorithm. Subsequently, the DCC is calculated based on the coordinates of the Cα atoms of the binding amino acids, and the DVO is calculated based on the volume of atoms contained in the binding amino acids.

#### 3.8.4. P2Rank

P2Rank [24] is a machine learning-based tool for fast and accurate prediction of ligand binding sites in protein structures. It initially generates a series of uniformly distributed points on the solvent-accessible surface of a protein and calculates feature descriptors for these surface points based on their local chemical environment. A random forest classifier is then used to predict the ligandicity score of each surface point. Points with high ligandicity scores are clustered into pockets for prediction. Finally, the pockets are ranked based on the sum of ligandicity scores of all points within each pocket.

## 4. Conclusions

Protein–ligand binding site prediction is a crucial component in structural biology and drug discovery, vital for understanding the molecular basis of biological functions and diseases. This study introduces PGpocket, a novel framework based on geometric deep learning, designed for accurate prediction of protein–ligand binding sites. By converting protein surfaces into point clouds and utilizing point cloud graph neural networks, PGpocket captures the intrinsic geometric and chemical properties of proteins at a fine-grained level. The model was comprehensively trained on the scPDB dataset and subsequently validated on two independent test sets, Coach420 and HOLO4K. Compared to existing methods, PGpocket demonstrates superior performance on these test sets, achieving a success rate of 58% on the Coach420 dataset and a success rate of 56% on the HOLO4K dataset. These results highlight the significant advancements made by PGpocket in the field of protein–ligand binding site prediction, showcasing its practicality and potential for future applications in drug design and structural biology.

There are several promising directions for future work. First, there is a need to enhance the interpretability of the model. This includes not only accurately predicting binding sites but also elucidating how these sites interact with small molecules from a biochemical perspective, providing insights that can be evaluated and validated by biochemists. Second, there is a need to develop models capable of predicting protein binding sites specific to different ligands. Current methods often identify binding sites on proteins without specifying the pockets that interact with specific small molecules. Third, PGpocket currently provides only the location of binding sites but does not specify the specific amino acids involved in the binding [10,27]. Therefore, in the future, we plan to improve the method to enable the prediction of binding amino acids. Finally, we intend to make the method available as a web server for users to access and utilize.

## Figures and Tables

**Table 1:** Summary of the performance of PGpocket and the baselines in scPDB dataset. Bold indicates the optimum value in each column.

Model	Number of Correctly Predicted Sites	Number of Binding Sites Not Identified	Success Rate	Avg DVO
COACH	298	34	0.564	0.42
Kalasanty	270	28	0.510	0.46
PUResNet	322	48	0.610	0.61
P2Rank	340	25	0.642	0.62
PGpocket	**350**	**13**	**0.662**	**0.65**

**Table 2:** Summary of the performance of PGpocket and the baselines in Coach420 and HOLO4K datasets. Bold indicates the optimum value in each column.

Dataset	Model	Number of Correctly Predicted Sites	Number of Binding Sites Not Identified	Success Rate (%)	Avg DVO
Coach420	COACH	159	28	50.1	0.28
	Kalasanty	162	26	51.0	0.30
	PUResNet	169	19	52.8	0.32
	P2Rank	175	20	55.2	0.34
	PGpocket	**184**	**13**	**57.8**	**0.35**
HOLO4K	COACH	2684	364	49.3	0.23
	Kalasanty	2776	325	51.0	0.21
	PUResNet	2994	428	54.0	0.25
	P2Rank	2918	298	53.6	**0.26**
	PGpocket	**3069**	**248**	**56.3**	0.24

**Table 3:** Summary of the performance of different model configurations in scPDB dataset. Bold indicates the optimum value in each column.

Model	Number of Correctly Predicted Sites	Number of Binding Sites Not Identified	Success Rate	Average Time (s)
All points	**356**	16	**0.674**	25.36
Downsampling but no feature aggregation	324	21	0.612	**12.56**
Downsampling with feature aggregation	350	**13**	0.662	14.65

## Data Availability

The datasets and code used in this work are available at https://github.com/zhaoyanpeng208/PGpocket, accessed on 3 August 2024.

## Supplementary Materials

The following supporting information can be downloaded at: https://www.mdpi.com/article/10.3390/ijms25179280/s1.
