# Supplementary material for: A Point Cloud Graph Neural Network for Protein–Ligand Binding Site Prediction
Source: Int J Mol Sci. 2024 Aug 27;25(17):9280. doi: 10.3390/ijms25179280 (PMC11394757; doi:10.3390/ijms25179280)
Supplement: Supplementary file 1 [file ijms-25-09280-s001.zip › ijms-3166291-supplementary.pdf]

Supplementary Information

A Point Cloud Graph Neural Network for Protein-Ligand Binding Site Prediction

Yanpeng Zhao [1,†], Song He [1,†], Yuting Xing [2], Mengfan Li [1], Yang Cao [1], Xuanze Wang [1], Dongsheng Zhao [1,*] and Xiaochen Bo [1,*]

1 Academy of Military Medical Sciences, Beijing 100850, China; zyp182531903@163.com (Y.Z.); hes1224@163.com (S.H.); li.mengfan@outlook.com (M.L.); cho.yang@foxmail.com (Y.C.); xuanze.wang@foxmail.com (X.W.)

2 Defense Innovation Institute, Beijing 100071, China; sukier1@126.com

* Correspondence: dszhao@bmi.ac.cn (D.Z.); boxiaoc@163.com or boxc@bmi.ac.cn (X.B.)

† These authors contributed equally to this work.

Supplementary Table S1. TP, FP, FN, and F1 score of PGpocket and the baselines in scPDB dataset. Bold indicates the optimum value in each column.

Model	TP	FP	FN	F1 score
COACH	298	197	34	0.721
Kalasanty	270	231	28	0.676
PUResNet	322	**159**	48	0.757
P2Rank	340	164	25	0.783
PGpocket	**350**	166	**13**	**0.796**

Supplementary Table S2. Success rate of different DCC values for PGpocket and the baselines on scPDB dataset.

DCC	PGpocket	Kalasanty	PUResNet	COACH	P2Rank
0	0	0	0	0	0
1	1.32	7.5	21.3	3.65	15.2
2	14.74	19.4	39.3	10.38	35.6
3	58.60	37.3	55.4	39.5	48.6
4	66.20	51	61	56.4	64.2
5	68.10	58.4	64.2	61.2	66.2
6	71.30	62.6	68.5	68.3	68.2
7	74.23	67.5	70.3	71.2	71.2
8	76.30	69.3	71.5	73.5	74.6
9	78.21	70.2	71.7	75.2	75.6
10	79.20	71.3	71.3	76.5	78.6
11	81.00	75.2	71.5	77.3	80.1
12	82.30	76.5	71.9	78.1	82.6
13	84.60	77.6	72.5	79.3	83.2
14	86.20	78.6	73.4	80.2	84.3
15	87.30	80.2	74.6	83.2	85.1
16	88.10	82.1	75.8	84.3	86.1
17	89.01	84.6	76.4	85.6	86.9
18	92.10	89.3	77.5	87.2	87.1
19	95.46	92.3	78.5	89.2	88.3
20	95.46	93.2	79.6	92.5	89.1

Supplementary Table S3. DVO values for PGpocket and the baselines on scPDB dataset.

DVO	COACH	Kalasanty	PUResNET	P2Rank	PGpocket
0	0	0	0	0	0
0.1	11	8	0	0	0
0.2	22	20	3	4	2
0.3	55	32	14	6	7
0.4	37	42	22	26	16
0.5	73	53	37	41	33
0.6	42	50	68	65	69
0.7	31	30	79	88	86
0.8	23	26	58	60	79
0.9	5	10	42	51	59
1	0	0	0	0	0

Supplementary Table S4. TP, FP, FN, and F1 score of PGpocket and the baselines in Coach420 and HOLO4K datasets. Bold indicates the optimum value in each column.

Dataset	Model	TP	FP	FN	F1 score
Coach 420	COACH	159	131	28	0.667
	kalasanty	162	130	26	0.675
	PUResNet	169	130	19	0.694
	P2Rank	175	123	20	0.710
	PGpocket	**184**	**121**	**13**	**0.733**
HOLO 4K	COACH	2684	2396	364	0.660
	kalasanty	2776	2343	325	0.675
	PUResNet	2994	**2022**	428	0.710
	P2Rank	2918	2228	298	0.698
	PGpocket	**3069**	2127	**248**	**0.721**

Supplementary Table S5. Success rate of different DCC values for PGpocket and the baselines on Coach420 dataset

DCC	PGpocket	Kalasanty	PUResNet	COACH	P2Rank
0	0	0	0	0	0
1	0.74	7.5	7.5	5.54	9.2
2	12.35	23.5	23.2	11.2	25.2
3	53.24	39.2	42.3	36.6	38.4
4	57.80	51	53	50.1	55.2
5	65.29	56.2	58.6	58.2	61.2
6	68.24	61.6	63.5	62.2	64.2
7	70.88	63.9	65.2	63.5	68.6
8	72.65	67.5	68.5	64.6	71.5
9	76.38	70.1	70.3	67.2	72.3
10	77.97	71.3	71.3	69.1	73.5
11	80.00	72.3	73.2	71.1	75.1
12	82.59	75.6	76.9	72.3	76.3
13	85.74	77.6	78.6	73.3	78.1
14	86.32	78.6	80.3	74.6	79.3
15	86.76	79.5	81.6	75.1	81.2
16	87.35	82.1	84.5	76.1	83.5
17	88.53	84.6	86.5	78.2	84.6
18	88.82	86.2	87.6	81.2	86.5
19	89.12	88.6	90.1	84.5	87.5
20	89.85	90.3	91.3	86.5	90.2

Supplementary Table S6. DVO values for PGpocket and the baselines on Coach420 dataset

DVO	COACH	Kalasanty	PUResNET	P2Rank	PGpocket
0	0	0	0	0	0
0.1	27	17	20	13	13
0.2	30	34	32	23	21
0.3	34	31	26	38	38
0.4	30	34	34	35	38
0.5	20	22	31	34	33
0.6	12	17	14	17	27
0.7	4	6	10	13	10
0.8	2	2	3	3	5
0.9	0	0	0	0	0
1	0	0	0	0	0

Supplementary Table S7. Success rate of different DCC values for PGpocket and the baselines on HOLO4K dataset

DCC	PGpocket	Kalasanty	PUResNet	COACH	P2Rank
0	0.00	0	0	0	0
1	0.74	7.0811083	6.2	1.35	8.1
2	23.35	17.794901	19.6	12.32	21.5
3	46.24	34.61952041	37.6	35	35.6
4	56.30	51	54	49.25	53.6
5	60.32	56.23881853	60.6	56.3	62.2
6	65.62	61.82051888	65.2	60.2	67.2
7	70.35	68.6286959	68.5	62.3	71.2
8	74.32	70.3	72.6	65.3	74.6
9	76.40	72.3	75.3	67.5	75.6
10	78.29	75.6	77.2	69.5	78.6
11	80.45	78.6	79.2	71.2	80.1
12	81.58	79.1	80.2	72.3	82.6
13	82.56	80.5	81.5	74.2	83.2
14	83.38	81	81.6	76.2	84.3
15	84.16	82.6	81.9	78.6	85.1
16	85.20	82.8	82.3	82.5	86.1
17	86.19	83.8	84.5	83.5	86.9
18	87.19	84.5	85.6	84.6	87.1
19	88.19	85.2	86.4	85.1	88.3
20	89.19	86.2	88.5	85.6	89.1

Supplementary Table S8. DVO values for PGpocket and the baselines on HOLO4K dataset

DVO	COACH	Kalasanty	PUResNET	P2Rank	PGpocket
0	0	0	0	0	0
0.1	225	319	383	371	431
0.2	798	924	678	638	764
0.3	1022	1015	852	788	872
0.4	516	444	612	622	606
0.5	113	65	337	350	279
0.6	11	10	109	121	91
0.7	0	0	19	23	22
0.8	0	0	5	6	5
0.9	0	0	0	0	0
1	0	0	0	0	0

Supplementary Table S9. TP, FP, FN, and F1 score of different model configurations in scPDB dataset. Bold indicates the optimum value in each column.

Model	TP	FP	FN	F1 score
All points	356	157	16	0.805
Downsampling but no feature aggregation	324	184	21	0.760
Downsampling with feature aggregation	350	166	13	0.796
